# Systemic vitamin intake impacting tissue proteomes

**DOI:** 10.1186/s12986-020-00491-7

**Published:** 2020-08-26

**Authors:** Heesoo Jeong, Nathaniel M. Vacanti

**Affiliations:** Division of Nutritional Sciences, Cornell University, Ithaca, NY USA

**Keywords:** Proteomics, Big data, Vitamin, Metabolism, Precision nutrition, Molecular nutrition

## Abstract

The kinetics and localization of the reactions of metabolism are coordinated by the enzymes that catalyze them. These enzymes are controlled via a myriad of mechanisms including inhibition/activation by metabolites, compartmentalization, thermodynamics, and nutrient sensing-based transcriptional or post-translational regulation; all of which are influenced as a network by the activities of metabolic enzymes and have downstream potential to exert direct or indirect control over protein abundances. Considering many of these enzymes are active only when one or more vitamin cofactors are present; the availability of vitamin cofactors likely yields a systems-influence over tissue proteomes. Furthermore, vitamins may influence protein abundances as nuclear receptor agonists, antioxidants, substrates for post-translational modifications, molecular signal transducers, and regulators of electrolyte homeostasis. Herein, studies of vitamin intake are explored for their contribution to unraveling vitamin influence over protein expression. As a body of work, these studies establish vitamin intake as a regulator of protein abundance; with the most powerful demonstrations reporting regulation of proteins directly related to the vitamin of interest. However, as a whole, the field has not kept pace with advances in proteomic platforms and analytical methodologies, and has not moved to validate mechanisms of regulation or potential for clinical application.

## Introduction

### Regulatory Mechanisms

Cellular metabolism is a system of chemical reactions in which cells harness the energy stored in the chemical bonds of substrate molecules to perform their biological functions, maintain homeostasis, or to synthesize building blocks for structural maintenance or cellular division. The kinetics of these reactions are dependent on the activity of the proteins which catalyze them; thus proteins are key modulators of metabolism.

Metabolic activity also exerts network control over itself by a diverse array of mechanisms which finely tune protein expression responses via nutrient sensing machineries [1]. Products or intermediates of a metabolic pathway can inhibit or activate metabolic enzymes; e.g. malate inhibits the succinate dehydrogenase complex [2] and fructose-2,6-bisphosphate activates phosphofructokinase [3]. The oxidative status of a cell can drive the directionality of redox reactions and impact abundances of redox reaction-catalyzing proteins; e.g. the KEAP1/NRF2 network responds to oxidative stress by upregulating expression of antioxidant-functioning proteins [4]. Splice-variant or isozyme expression can impact relative pathway utilization at metabolic network nodes; e.g. splice variants and isozymes of pyruvate and lactate dehydrogenase respectively impact the bridge between glycolysis and the tricarboxylic acid (TCA) cycle [5, 6]. Additionally, local metabolite concentrations and thermodynamics can dictate the directionality of reactions catalyzed by compartment-specific isozymes; e.g. reductive activity of isocitrate dehydrogenase can be confined to the cytosol-specific isozyme [7]. The impacts of the above-mentioned regulations are closely monitored by nutrient sensing proteins which initiate molecular events altering protein activation and expression; e.g. serine/threonine kinase 11, AMP-activated protein kinase, mammalian target of rapamycin 1, and sterol regulatory element-binding protein 1 are part of overlapping protein networks that orchestrate protein-expression and post-translational modification responses to nutrient availability [8, 9]. Considering that many metabolic enzymes do not function in isolation and, as detailed in the sections that follow, require vitamin cofactors to stabilize intermediates, donate/accept electrons, shuttle substrates, and hold reactants in close proximity; vitamin status is a critical consideration when examining protein-mediated regulation of metabolism and the impacts of metabolism on protein expression.

In addition to their potential regulatory roles as cofactors, vitamins orchestrate other direct or indirect mechanisms influencing protein abundance. Retinoic acid (vitamin A) interacts with nuclear receptors impacting gene transcription [10], ascorbic acid (vitamin C) impacts oxidative status and associated protein networks [11] and is reported to exhibit epigenetic regulation over protein expression [12], vitamin D regulates calcium signaling machinery, activates nuclear receptors, and exerts hormonal regulation over protein expression [13, 14], and niacin (vitamin B_3_) and biotin (vitamin B_7_) can be incorporated as post-translational modifications impacting protein function [15, 16].

**Fig. 1:**
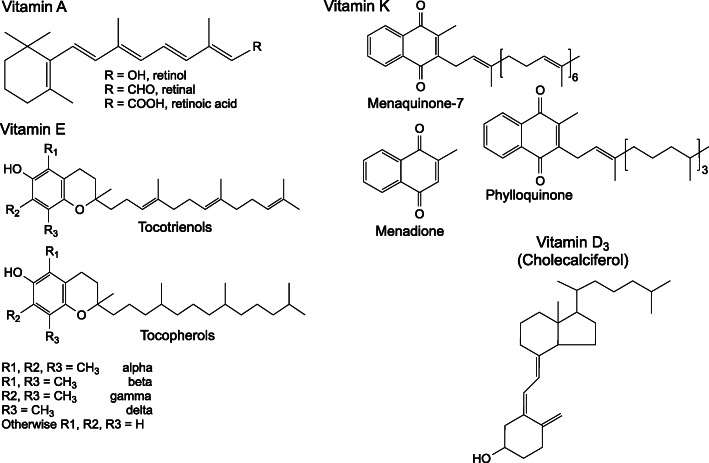
Fat soluble vitamin structures

**Fig. 2:**
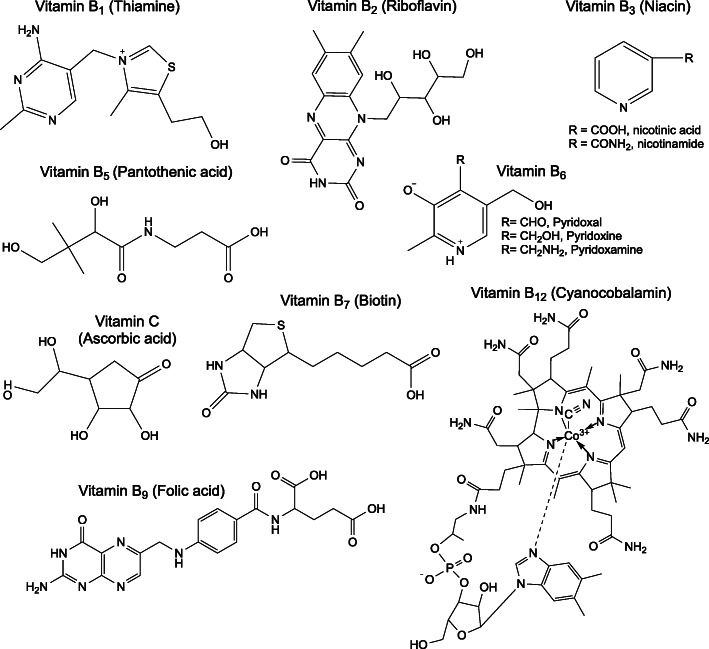
Water soluble vitamin structures

**Fig. 3:**
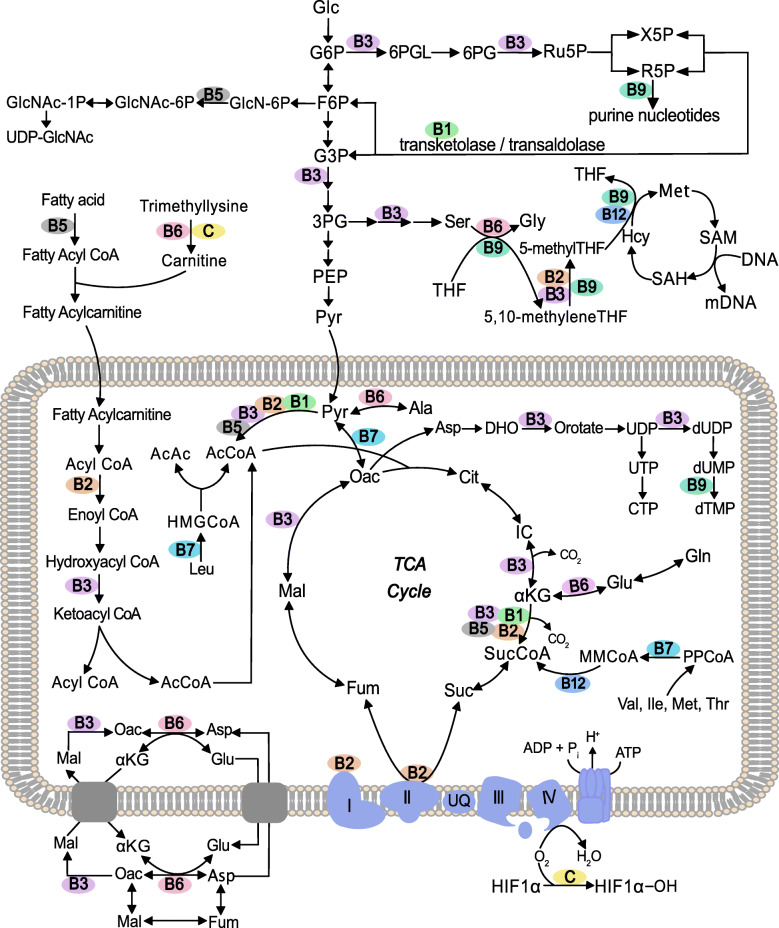
Schematic of vitamin involvement in reactions of central carbon metabolism. The depicted lipid bilayer represents the inner mitochondiral membrane. Abbreviations defined in the abbreviations section. Vitamins specified by alphanumeric designations

Herein, studies on systemic intake (dietary, injection, oral gavage) of vitamins and their impacts on tissue proteomes are examined, and their contributions to unraveling vitamin-based regulation of protein expression and tissue function are explored. The current work is intended to provide background information to understand each vitamin’s (Figs. 1 and 2) molecular functions and highlight its role as a cofactor or substrate in the reactions of central metabolism (Fig. 3, Tables S1, S2, S3, S4, S5, S6, S7, S8, S9, S10, S11, S12 and S13). Finally, this work is intended as a resource for identifying regulation of proteins related to vitamin metabolism in published works. The public domain of proteomic data sets is ever expanding, but is rarely searched for effects related to vitamin metabolism. To that end, all proteins are specified by their HUGO Gene Nomenclature Committee (HGNC) gene symbol, or the HGNC gene symbol of the human ortholog when identified in another species, and proteins requiring a vitamin as a cofactor or substrate are tabulated (Tables S1, S2, S3, S4, S5, S6, S7, S8, S9, S10, S11, S12 and S13).

### Proteomics Platforms

Proteomics platforms of the discussed studies are provided to place them on a technological timeline. Platforms are described with the terms: orbitrap, QTOF (quadrupole time-of-flight), triple-TOF (triple – time of flight), QQQ (triple-quadrupole), 2DGE-MS (two-dimensional gel electrophoresis – mass spectrometry), and 2DGE. In brief, orbitrap platforms are the workhorses of modern proteomics because their high achievable mass resolutions combined with high sensitivity are best suited for maximizing the number of proteins identified in a complex sample [17, 18]; though QTOF and triple-TOF instruments, capable of maintaining mass resolution at higher scan speeds [19], hold a substantial influence in this arena. Within the categories of orbitrap, QTOF, and triple-TOF, there are major technological advances not discussed here. QQQ platforms are best suited for quantifying a pre-determined list of proteins. Lower scan speeds and mass resolution render them less capable than orbitrap, QTOF, or triple-TOF systems for non-targeted applications [17]. Advances in nano-flow liquid chromatography coupled directly to mass spectrometry have improved proteomic depth by orders of magnitude over that achievable by 2DGE-MS, where the upstream selection of protein spots predates the modern definition of non-targeted proteomics. Similarly, identifying differentially intense protein spots using 2DGE alone is considered an important milestone in the development of proteomics; but is rarely discussed outside the topic of the field’s history.

## Vitamin Regulation of Tissue Proteomes

### Vitamin A

Vitamin A exists in alcohol, aldehyde, acid, and ester forms known as retinol, retinal, retinoic acid, and retinyl esters respectively (Fig. 1) [20]. Several carotenoids are precursors to vitamin A including α- and β-carotene [21]. β-carotene is converted to two molecules of retinal by beta carotene oxygenases (*BCO1* or *BCO2*) [22]. Retinal is an important component of rhodopsin (*RHO*), a protein in rod cells responsible for detecting low levels of light [23]. Thus night blindness is telltale characteristic of vitamin A deficiency [24]. Retinoic acid serves as a signaling molecule, acting through nuclear retinoic acid (*RARA*, *RARB*, *RARG*) and retinoid X (*RXRA*, *RXRB*, *RXRG*) receptors which regulate growth and differentiation [25, 26]. Cellular and organismal trafficking of vitamin A is dependent on retinol/retinoic acid binding proteins (*RBP* family, *CRABP1*, *CRABP2*) and retinol esterification via lecithin retinol acyltransferase (*LRAT*) [27]. Retinal is oxidized to retinol via aldehyde dehydrogenases (*ALDH* family) and retinol is oxidized to retinoic acid by retinol dehydrogenases (*RDH* and *DHRS* families) [28]. In addition to inducing night blindness, vitamin A deficiency adversely impacts cellular growth, bone development, and antibody-based immune responses [29].

In an orbitrap-based study of mouse embryo heads, toxic levels of prenatal retinoic acid exposure intended to model an established risk factor for craniofacial birth defects are reported to induce abundance alterations in proteins associated with craniofacial development and neural crest processes [30]. In a parallel triple-TOF-based study of gerbil plasma and 2DGE-MS-based study of gerbil liver and white adipose tissue, a few dozen protein abundances linked to a handful of biological processes are reported to respond to dietary retinol, β-carotene, lutein, or lycopene; though process or pathway enrichment analyses are not reported. As the authors discuss, plasma was not depleted of common highly abundant proteins upstream of analysis by mass spectrometry which are known to adversely impact data quality [31]. In an orbitrap-based study of plasma from Nepalese children, dozens of proteins are associated with circulating carotenoid abundances; potentiating development of low-cost antibody-based tests for carotenoid deficiencies [32]. A pair of 2DGE-MS-based studies link tissue function to protein abundance responses to vitamin A status in mice brains [33] and bovine muscle [34].

### Vitamin B_1_

Thiamine (vitamin B_1_) is composed of linked pyrimidine and thiazole rings decorated with methyl, amine, and alkyl-hydroxyl functional groups (Fig. 2) [35]. Thiamine is transported through the plasma membrane via thiamine transporters (*SLC19A2* and *SLC19A3*) [36] and then twice phosphorylated on the alkyl-hydroxyl functional group by thiamine pyrophosphokinase (*TPK1*), rendering it active as thiamine diphosphate (TDP) [35]. TDP is a cofactor for enzymes catalyzing the oxidative decarboxylation of ketoacids including the pyruvate dehydrogenase complex (*PDHA*, *PDHB*, *PDHX*, *DLAT*, *DLD*), the oxoglutarate dehydrogenase complex (*OGDH*, *DLST*, *DLD*), and the branched chain keto acid dehydrogenase complex (*BCKDHA*, *DBT*, *DLD*) [37]. It is also a cofactor for transketolase (*TKT*) in the non-oxidative branch of the pentose phosphate pathway [38]. Independent from its role as a cofactor, thiamine is believed to regulate ion transport activity in the nervous system [39].

Vitamin B_1_ deficiency is marked by a broad range of neurological, respiratory, and cardiovascular pathophysiologies and is termed beriberi. Symptoms of beriberi are difficult to directly link to the molecular functions of vitamin B_1_ [40].

In a 2DGE-MS-based study of type 2 diabetic and healthy control subjects, authors report treatment with thiamine reduces albumin (*ALB*) abundance in urine; indicating the vitamin serves a protective role of kidney function [41]. In a QTOF-based study of rat thalami under thiamine deficiency, glyceraldehyde-3-phosphate dehydrogenase (*GAPDH*) is the most up-regulated protein (50 fold) while regulated proteins are most enriched in the synaptic vesicle cycle pathway (according to the KEGG database). Proteomic changes are accompanied by diminished performances on cognitive tests [42].

### Vitamin B_2_

Riboflavin (vitamin B_2_) is composed of an isoalloxazine ring and a bound ribitol (Fig. 2) [43]. It is activated by riboflavin kinase (*RFK*), forming flavin mononucleotide (FMN); and by flavin adenine dinucleotide synthase 1 (*FLAD1*), forming flavin adenine dinucleotide (FAD) [44]. Bound FMN or FAD serves as an electron carrier for redox-reaction-catalyzing proteins (flavoproteins) including the succinate dehydrogenase complex (*SDHA*, *SDHB*, *SDHC*, *SDHD*), the pyruvate dehydrogenase complex (*PDHA*, *PDHB*, *PDHX*, *DLAT*, *DLD*), acyl-CoA dehydrogenases (*ACAD*s), and methylene tetrahydrofolate reductase (*MTHFR*) [45].

Riboflavin deficiency in humans predominantly occurs in combination with that of other nutrients. However animal studies link it to impaired fetal and intestinal development [46, 47], iron absorption [48], and lipid metabolism [49, 50].

In a QTOF-based study of duck livers, riboflavin deficiency is accompanied by a reduced abundance of small-chain-specific acyl-coenzyme A dehydrogenases (*ACADs*), for which riboflavin serves as a cofactor, and concordant elevation of hepatic small chain fatty-acid lipid content. Dramatic decreases in protein abundance are reported for *INPP1* (involved in inositol signaling), *THRSP* (purported regulator of lipid metabolism), *BDH2* (a regulator of lipid metabolism), *FXN* (involved in mitochondrial iron-sulfur complex assembly), and *NDUFS1* (a subunit of electron transport chain complex I) [51]. In a QTOF-based study of maternal riboflavin deficiency, reductions in fetal duck hepatic TCA cycle, beta-oxidation, and electron transport chain proteins are reported, with *IDH3A* being the lone member of these pathways whose abundance increases [52].

### Vitamin B_3_

Niacin (vitamin B_3_) is inclusive of nicotinic acid and nicotinamide (Fig. 2) which are converted to their mononucleotide forms by nicotinate phosphoribosyltransferase (*NAPRT*) and nicotinamide phosphoribosyltransferase (*NAMPT*) respectively [53]. Both forms of the mononucleotide are subsequently converted to their adenosine dinucleotide forms by nicotinamide/nicotinic acid mononucleotide adenylyltransferases (*NMNAT1*, *NMNAT2*, *NMNAT3*). Nicotinamide adenine dinucleotide (NAD) is a cofactor form of the vitamin whereas nicotinic acid dinucleotide is subsequently converted to NAD by NAD synthase (*NADSYN1*) [54]. NAD is reduced to NADH by oxidative reactions of glycolysis, the TCA cycle, and β-oxidation; and subsequently serves as a redox equivalent carrier to the electron transport chain [55] and to regenerate reduced ascorbic acid (vitamin C) [56], glutathione [57], and thioredoxin [58]. NAD can also be phosphorylated by NAD kinases (*NADK*, *NADK2*) to form a distinct redox shuttling cofactor, NADP [59]. NADP is reduced by reactions in the oxidative pentose phosphate pathway (*G6PD*, *PGD*) and other enzymes (e.g. *ME1*, *ME3*, *IDH1*, *IDH2*) to NADPH. NADPH provides reducing equivalents for biosynthetic reactions in fatty acid, cholesterol, and deoxyribonucleotide synthesis [60]. Outside its role as a reducing equivalent shuttle, NAD provides adenine dinucleotide phosphate (ADP) ribose for synthesis of the second messenger, cyclic adenosine monophosphate (cAMP), via the activity of adenylate cyclases (*ADCY* family) [61]. NAD also provides ADP-ribose and poly-ADP-ribose for post translational modifications of proteins via activity of ADP-ribosyl transferases (*ART* family) and ADP ribose polymerases (*PARP* family) [62, 63]. cAMP and protein (poly)ADP-ribosylation are important mediators of cell signaling and protein expression [64]. Niacin is synthesized from tryptophan, but in small quantities relative to a healthy dietary intake [65]. Deficiency, known as pellagra, is marked by dermatitis and severe gastrointestinal/neurological pathophysiologies which are fatal if untreated [66]. No proteomic studies on systemic intake of vitamin B_3_ were found at the time of writing this review.

### Vitamin B_5_

Pantothenic acid (vitamin B_5_) is composed of a molecule of pantoic acid bound to β-alanine (Fig. 2) [67]. Its primary metabolic function is as an acyl-carrier [68]. Pantothenic acid is a substrate in the first reaction of coenzyme A (CoA) biosynthesis catalyzed by pantothenate kinases (*PANK1*, *PANK2*, *PANK3*, *PANK4*) [69]. CoA is a substrate for enzymes catalyzing the oxidative decarboxylation of ketoacids including the pyruvate dehydrogenase complex (*PDHA*, *PDHB*, *PDHX*, *DLAT*, *DLD*), the oxoglutarate dehydrogenase complex (*OGDH*, *DLST*, *DLD*), and the branched chain keto acid dehydrogenase complex (*BCKDHA*, *DBT*, *DLD*) [70–72]. Acyl species are activated by conjugation with CoA and are substrates in or products of glycolysis, the TCA cycle, fatty-acid synthesis/β-oxidation, cholesterol synthesis, ketogenesis, branched-chain amino acid catabolism, and protein acetylation/O-GlcNAcylation [73]. Finally, 4’-phosphopanthetheine (product of *PANK* proteins’ activities) is a cofactor of the acyl carrier protein domain of fatty acid synthase (*FASN*) [74]. Vitamin B_5_ deficiency is rare and usually accompanied by that of other nutrients [75]. Burning of the feet and numbness in the toes is a characteristic manifestation along with variety of other symptoms [76]. No proteomic studies on systemic intake of vitamin B_5_ were found at the time of writing this review.

### Vitamin B_6_

Vitamin B_6_ has aldehyde, alcohol, and amine forms (Fig. 2); of which the phosphorylated aldehyde form (pyridoxal phosphate) acts as a cofactor to over 100 enzymes [77]. All three forms of vitamin B_6_ are phosphorylated by pyridoxal kinase (*PDXK*) [78]. Both the phosphorylated alcohol and amine forms (pyridoxine phosphate and pyridoxamine phosphate) are converted to pyridoxal phosphate by pyridoxine phosphate oxidase (*PNPO*) [79]. Pyridoxal phosphate is a cofactor for enzymes catalyzing decarboxylase reactions in gamma-aminobutyric acid (*GAD1*, *GAD2*) [80] and serotonin/dopamine biosynthesis (*DDC*) [81]; as well as for enzymes catalyzing transamination reactions (e.g. *GOT1*, *GOT2*, *GPT*, *GPT2*) [82], cysteine synthesis (*CTH*) [83], heme synthesis (*ALAS1*, *ALAS2*) [84], carnitine synthesis (3-hydroxy-6-N-trimethyllysine aldolase, gene unidentified) [85], niacin synthesis (*KYNU*) [86], and sphingolipid synthesis (*SPTLC1*, *SPTLC2*) [87]. Pyridoxal phosphate is also an important cofactor for enzymes of one-carbon metabolism (*SHMT1* and *SHMT2*) [88] and glycogen catabolism (*PYGL* and *PYGM*) [89]. Vitamin B_6_ deficiency is rare because of its availability in many foods, and pathophysiologies can be diverse [90].

In a triple-TOF-based study of streptozotocin-induced diabetic rat hippocampi, pyridoxamine treatment prevented long-term recognition memory impairment and regulated protein abundances in a number of diverse pathways; notably upregulating half of the proteins involved in ubiquinol biosynthesis [91]. In a 2DGE-MS-based study of mice hippocampi, the abundances of phosphoglycerate mutase (*PGAM1*) and cannabinoid receptor-interacting protein 1 (*CNRIP1*) are reported to be elevated/reduced, respectively, upon administration of pyridoxine. Proteomic changes are accompanied by improved novel object recognition [92].

### Vitamin B_7_

Biotin (vitamin B_7_) is composed of a fused-ring structure bound to a valeric acid side chain (Fig. 2) [93]. It is transported across the plasma membrane by the sodium-dependent solute carriers *SLC5A6* and *SLC19A3* [94, 95]. As a cofactor/post-translational modification, biotin covalently binds lysine residues [96]. It is a cofactor for pyruvate carboxylase (*PC*), acetyl-CoA carboxylase (*ACACA*), propionyl-CoA carboxylase (*PCCA*), and the methylcrotonyl-CoA carboxylase complex (*MCCC1*, *MCCC2*) [97]. Histones are also biotinylated, regulating gene expression [98]. The post-translational modification occurs via the activity of holocarboxylase synthetase (*HLCS*) [99].

Biotin deficiency is rare and has wide ranging pathophysiologies. Eating raw egg whites can prevent its absorption (leading to deficiency) because of its affinity for avidin, a chemical in egg whites that is denatured upon cooking. This observation led to the vitamin’s eventual discovery [100]. No proteomic studies on systemic intake of vitamin B_7_ were found at the time of writing this review.

### Vitamin B_9_

The term folate (vitamin B_9_) is inclusive of a group of compounds composed of a pteridine ring linked to para-aminobenzoic acid with a mono- or polyglutamate tail (Fig. 2) [101]. In its reduced form (tetrahydrofolate), a one-carbon unit cross-links (as CH or CH_2_) amine groups on the ring structure and aminobenzoic acid, or binds the secondary amine (as a formyl group) on the aminobenzoic acid group [102, 103]. This one-carbon unit is utilized in the synthesis of purines and thymidine, conversion of homocysteine to methionine, interconversion of serine and glycine, and catabolism of histidine; reactions collectively termed one-carbon metabolism [104, 105]. At the cellular level, one-carbon metabolism is tightly regulated by compartmentalization [104, 106, 107] while whole-body folate homeostasis is predominantly maintained by the liver through the enterohepatic cycle [108].

Folate deficiency induces megaloblastic macrocytic anemia and fetal neural tube defects, purportedly via its adverse impact on nucleotide synthesis [109, 110]. Low intake of folate is also linked to cardiovascular disease [111, 112], neurodegenerative disease [113, 114], Alzheimer’s disease [115, 116] and cancer [117–119].

In an orbitrap-based study of follicle fluid of women undergoing in vitro fertilization, the folate supplemented group is reported to have elevated abundances of apolipoproteins from high density lipoproteins and reduced reactive protein c (*CRP*). The study is performed on women who did not become pregnant [120]. In a QTOF-based study of a folate-deficiency-induced intestinal neoplasia mouse model, the combinatorial impacts of folate deficiency and methylene tetrahydrofolate reductase heterozygous deletion (*mthfr*^+/-^) are reported to impact protein abundances spanning diverse cellular functions. However 40% of samples are discarded as outliers and the simultaneous examination of *mthfr*^+/-^ and dietary folate deficiency does not allow proteomic adaptations to be attributed to either in isolation [121]. In a 2DGE-MS-based study of adult rats, aortic calmodulin (*CALM1*, calcium signaling) protein abundances are positively correlated with folate dose while abundances of triose phosphate isomerase (*TPI1*, glycolysis), transgelin (*TAGLN*, cytoskeleton), and glutathione s-transferase alpha 3 (*GSTA3*, reductive detoxification) respond inversely [122]. In an 2DGE-MS-based study of rat livers, *PRDX6* and *GPX1* are reported to be elevated while cofilin (*CFL1*) is reported to be depleted under folate deficiency [123]. Other studies report protein abundance differences due to folate intake in rat urinary exosomes (QQQ-based) [124], human plasma (2DGE-MS) [125], fetal brain tissue from pregnant mice fed ethanol (2DGE-MS) [126], pregnant rat livers (2DGE-MS) [127], fetal rat livers (2DGE-MS) [128], adult rat livers and brains (2DGE-MS) [129], and livers of piglets born to folate deficient mothers (2DGE-MS) [130].

### Vitamin B_12_

Cobalamin (vitamin B_12_) encompasses a group of molecules with four linked pyrrole ring derivatives (forming a corrin ring) and a cobalt atom bound at the center of the corrin ring. The cobalt atom also binds a 5,6-dimethylbenzimidazole nucleotide and a functional group (Fig. 2) [131]. The identity of the functional group distinguishes the vitamin B_12_ compounds as cyanocobalamin, hydroxycobalamin, hydrocobalamin, nitrocobalamin, 5’-deoxyadenosylcobalamin (also called adenosylcobalamin), and methyl cobalamin [132, 133]. Methylcobalamin serves as a coenzyme in the conversion of homocysteine to methionine by methionine synthase (*MTR*) in the cytosol [134] and adenosylcobalamin is required for conversion of L-methylmalonyl-CoA to succinyl-CoA by methylmalonyl-CoA mutase (*MUT*) in mitochondria [135].

Vitamin B_12_ deficiency is closely related to folate deficiency and can lead to megaloblastic anemia by impairment in the activity of methionine synthase (*MTR*) [109]: 5-methyl tetrahydrofolate cannot be converted to one-carbon donors required for purine and thymidine synthesis without vitamin B_12_ as a cofactor, thus interfering with DNA synthesis and erythrocyte production [136]. Vitamin B_12_ deficiency is also linked to neurological disorders independent of anemia [137].

Ruoppolo and colleagues performed a 2DGE-MS-based study of lymphocytes isolated from methylmalonic acidemia with homocystinuria, cobalamin deficiency type C (MMACHC) patients (an inborn error in metabolism marked by inactivity of the *MMACHC* gene product) receiving a standard treatment of hydroxycobalamin, betaine, folate, and carnitine. Protein products of *ME2*, *GLUD1*, and *GPD2*, genes involved in anaplerosis and redox equivalent shuttling, are up-regulated while variant 2 of protein pyruvate kinase muscle isozyme (*PKM*) and lactate dehydrogenase B (*LDHB*) are down-regulated relative to lymphocytes isolated from healthy control donors [138]. In a 2DGE-based study of adult rat cerebral spinal fluid, protein abundance shifts are reported to peak after several months on a cobalamin deficient diet (modest shifts) or after a total gastrectomy (more severe shifts), and return to near control values at later time points [139]. In a 2DGE-MS-based study, glutathione s-transferase P (*GSTP1*) abundances are diminished and glutathione peroxidase 1 (*GPX1*) abundances are elevated in rat pup kidneys under maternal vitamin B_12_ deficient and maternal folate deficient conditions [140]; suggesting maternal dietary intake of these vitamins impacts offspring kidney redox homeostasis mechanisms. In a similar 2DGE-MS-based study of maternal vitamin B_12_ deficiency, the same group reports that several dozen rat kidney pup proteins revert to control levels upon administration of vitamin B_12_ at birth. Additionally, diminished abundance of beta-oxidation proteins in kidneys of pups born to vitamin B_12_ deficient mothers is accompanied by elevated *PPARA* [141], a positive regulator of fatty acid oxidation, suggesting attempted compensation at the cellular level.

### Vitamin C

Vitamin C (ascorbic acid) is absorbed at the brush-border and distributed to cells throughout the body by the sodium-dependent plasma membrane solute carriers *SLC23A1* and *SLC23A2* [142]. The oxidized form of vitamin C (dehydroascorbate) is also transported via plasma membrane glucose transporters *SLC2A1*, *SLC2A3*, and *SLC2A4* (also known as *GLUT1*, *GLUT3*, and *GLUT4*) [143] and reduced intracellularly to ascorbic acid by glutathione [144] and the activity of thioredoxin reductases (*TXNRD1*, *TXNRD2*, or *TXNRD3*) [145].

Vitamin C is a cofactor in the function of prolyl and lysyl hydroxylases, which consume oxygen and alpha-ketoglutarate to form the hydroxylated amino acid residue and succinate [146]. The Fe^2+^ of these enzymes is restored from Fe^3+^ by oxidation of vitamin C [147]. In the presence of oxygen, prolyl hydroxylases (*EGLN1*, *EGLN2*, *EGLN3*; also known as *PHD2*, *PHD1*, *PHD3* respectively) hydroxylate the *HIF1A* protein; providing a necessary signal for its degradation and preventing a hypoxic response at the cellular level [148]. Prolyl and lysyl hydroxylase activities are also necessary for post-translational modifications to form functional collagen [149]. Lysyl hydroxylases include *PLOD1*, *PLOD2*, and *PLOD3* [150]. Vitamin C serves a nearly identical function in reducing Fe^3+^ as a cofactor for trimethyllysine dioxygenase (*TMLH*), which catalyzes the first reaction in carnitine biosynthesis [151]. Carnitine is essential for fatty acid catabolism in the mitochondria as only fatty acyl carnitines formed via the activity of carnitine palmitoyl transferases *CPT1A*, *CPT1B*, and *CPT1C* cross the inner mitochondrial membrane through the solute carrier *SLC25A20* [152]. Vitamin C similarly serves as a cofactor for tyrosine hydroxylase (*TH*), which catalyzes the first reaction in catecholamine (e.g. dopamine, epinephrine, and norepinephrine) synthesis [153]. Additionally, vitamin C serves and as a general antioxidant [154]. Vitamin C deficiency leads to the condition known as scurvy with symptoms largely attributed to malformed connective tissue due to improperly folded collagen [155].

In a orbitrap-based study on a pig model of hemorrhagic shock, vitamin C administration is reported to impact plasma protein abundances in the complement pathway and those in poly-trauma related processes; including the stabilization of *ADAMTS13* abundance, an important regulator of clot formation [156]. An orbitrap-based study of endoplasmic reticulum enriched fractions of livers in Werner syndrome mouse models identifies around a dozen proteins whose abundances are impacted by administration of vitamin C [157]. A QTOF-based study of zebrafish reports upregulation of glutamate dehydrogenase (*GLUD1*) and downregulation of pyruvate kinase muscle isozyme (*PKM*) upon administration of vitamin C in a vitamin E deficient background [158]. In a QQQ-based study of human plasma, ascorbic acid concentration is reported to be inversely related to vitamin D binding protein (*GC*) abundance [159]. 2DGE-MS-based studies identify protein abundance regulations in mouse models of sarcoma metastases in the liver [160] and tumor nodules of adenocarcinoma due to administration of vitamin C [161]. Another 2DGE-MS-based study reports polypeptide abundance shifts in hemodialysis patient plasma upon vitamin C supplementation [162].

### Vitamin D

Vitamins D_2_ and D_3_ are respectively distinguished by their ergosterol and cholesterol backbones [163]. Though only vitamin D_3_ is synthesized in animals, both can be converted to active forms. Exposure of 7-dehydrocholesterol (an intermediate in cholesterol synthesis) to ultra-violet radiation in the skin and subsequent isomerization produces cholecalciferol (vitamin D_3,_ Fig. 1) [164]. Whether 7-dehydrocholesterol is derived from cholesterol via activity of 7-dehydrocholesterol reductase (*DHCR7*) or synthesized de novo in the skin is disputed [165]. 7-dehydrocholesterol is successively hydroxylated by activity of cytochrome p450 enzymes (e.g. *CYP2R1* and *CYP27B1*) in the liver and kidney to its active 1,25-(OH)_2_ cholecalciferol [1,25(OH)_2_D_3_] form [166]. Transport of vitamin D and its metabolites occurs bound to vitamin D binding protein (*GC*) [167]. Ergocalciferol is the vitamin D_2_ equivalent of cholecalciferol and is activated analogously [168].

1,25(OH)_2_D_3_ influences cellular function via nuclear receptor-dependent and nuclear receptor-independent mechanisms. The former involves 1,25(OH)_2_D_3_-bound vitamin D receptor (*VDR*) forming a heterodimer complex with a retinoid X receptor (*RXRA*, *RXRB*, *RXRG*) and subsequently binding vitamin D response elements regulating transcription of genes largely involved modulating calcium and phosphorous transport [169] and maintaining homeostasis by regulating their absorption in the kidneys, intestines, and bones [170, 171]. The rapid-onset extracellular impacts (nuclear receptor-independent) of 1,25(OH)_2_D_3_ are mediated by a membrane-associated rapid response steroid binding protein, identified as *PDIA3* [172], and diversely impact cell growth, survival, and immune response [173].

Deficiency in vitamin D impairs bone mineralization causing rickets in infants/children and osteomalacia in adults [174]. Vitamin D deficiency is also linked to cardiovascular diseases [175, 176], cancer [177, 178], neurological impairments [179, 180] and autoimmune diseases [181, 182]; though underlying mechanisms are not completely understood.

In an orbitrap-based study of mouse fetal and postnatal lung tissue, maternal vitamin D deficiency is reflected in total proteome adaptations which are unexpectedly strongest at postnatal day 7 opposed to fetal time points. Impacted proteins include several associated with lung development [183]. An orbitrap-based study of a mouse brain tissue model of remyelination in multiple sclerosis reports calcium binding protein abundances to be upregulated upon treatment with 1,25(OH)_2_D_3_, consistent with the vitamin's regulatory role over calcium absorption [184]. In an orbitrap-based study of serum from overweight adults, vitamin D deficiency is reported to differentially affect abundances of proteins related to blood coagulation in males and females. However, abundances of these proteins are likely impacted by the production of serum from whole blood. The authors report quantifying 1,841 proteins (Table 1); an impressive analytical depth for serum [188]. In a 2DGE-based study, vitamin D deficient children are reported to have diminished serum abundances of adiponectin (*ADIPOQ*) [189]. In a separate 2DGE-based study, the same group reports fetuin-b (*FETUB*) to be elevated in the plasma of obese vitamin D deficient children compared with their vitamin D sufficient counterparts [190]. However the authors do not directly identify *FETUB* and rely on comparison of their findings to those of another study [191]. Two 2DGE-MS-based studies, of rat left ventricular and aortic tissue, identify proteins whose abundances respond upon inducing arterial calcification or atherosclerosis by co-administration of vitamin D_3_ with nicotine or a high cholesterol diet respectively [192, 193]. Two studies (2DGE-MS and 2DGE-based respectively) examine the impacts of vitamin D deficiency on the rat brain proteome. The former reports the progeny of vitamin D deficient mothers to have diminished abundances of ATP synthase β (*ATPB*) and enolase 2 (*ENO2*) in both the cortex and hippocampus, and diminished calmodulin (*CALM1*) in the hippocampus amongst a variety of other regulated proteins [194]. The latter finds low vitamin D diets to be accompanied by diminished cortical abundances of three glycolytic enzymes: triose phosphate isomerase (*TPI1*); phosphofructokinase, platelet (*PFKP*); and pyruvate kinase, muscle (*PKM*) [195].

**Table 1:** Summary of key findings

Vitamin	Key Findings
Vitamin A	Creation of a model of craniofacial disorders induced by prenatal retinoic acid exposure is reported to impact protein abundances whose functions are associated with neural crest processes [30]. Plasma carotenoids abundances are reported to be associated with plasma proteins of diverse functions in Nepalese children, potentiating development of inexpensive assays to predict carotenoids deficiency [32].
Vitamin B_1_	Treatment with thiamine is presented as a potential strategy to improve kidney function in type 2 diabetic patients [41]. Thiamine deficiency is reported to impact cognition in rats [42].
Vitamin B_2_	Dietary and maternal dietary riboflavin are reported to impact the machineries of lipid metabolism and fetal lipid metabolism in ducks [51, 52].
Vitamin B_6_	A study of a rat model of diabetes reports treatment with pyridoxamine to impact abundances of proteins involved in synaptic plasticity in hippocampi and to have protective effects on long-term memory [91].
Vitamin B_9_	Folate supplementation in women undergoing in vitro fertilization is reported to increase abundances of apolipoproteins of high-density lipoproteins in monofollicular fluid [120].
Vitamin B_12_	Maternal cobalamin deficiency is reported to impact abundances of proteins related to lipid metabolism in the offspring kidneys of rats [141].
Vitamin C	Treatment with ascorbic acid is reported to impact plasma abundances of proteins involved in the complement pathway and regulation of clot formation in a pig model of hemorrhagic shock [156].
Vitamin D	Maternal vitamin D deficiency is reported to impact abundances of proteins involved in mouse neonatal lung development during alveolar development stages without affecting gross lung structure [183]. Treatment with 1,25(OH)_2_D_3_ is reported to increase abundances of proteins involved in calcium homeostasis in a mouse brain model of remyelination [184].
Vitamin E	A quantitative model based on plasma protein abundances is reported to predict plasma α-tocopherol status, potentiating the development of an inexpensive assay to detect α-tocopherol deficiency [185]. A study of a mouse model of Alzheimer’s disease reports treatment with the tocotrienol-rich fraction of palm oil reduces the abundance of amyloid beta A4 protein, the primary component of amyloid plaques, in hippocampi [186].
Vitamin K	A quantitative model based on five plasma protein abundances is reported to predict vitamin K deficiency with moderate accuracy [187].

### Vitamin E

Members of the vitamin E class of molecules all contain fused phenyl and chromanol rings linked to a 16-carbon side-chain [196]. Methyl group placement on the phenyl ring dictates α, β, γ, and δ designation while side-chain saturation state distinguishes tocopherols from tocotrienols (Fig. 1). Furthermore, all forms of vitamin E have three chiral centers resulting in 8 stereoisomers [197]. RRR α-tocopherol is the most biologically active form, likely due to specificity of α-tocopherol transfer protein (*TTPA*) whose binding is necessary for packaging and transport to tissues from the liver [198]. α-tocopherol primarily localizes to membranes (i.e. plasma, endoplasmic reticulum, and mitochondrial) and functions as an antioxidant for unsaturated, lipid-bound fatty acids [196]. Α-tocopherol also has non-antioxidant signal-transduction functions impacting a broad range of cellular activities [199].

Vitamin E deficiency is rare due to the availability of the vitamin in the diet [200], though it may be caused by a genetic defect in α-tocopherol transfer protein (*TTPA*) and diseases associated with fat malabsorption [201, 202]. Severe vitamin E deficiency can result in hemolytic anemia, neurological disorders, and ataxia [201, 203–205].

An orbitrap-based study of plasma from undernourished Nepalese children reports plasma α-tocopherol concentration to be positively correlated with abundances of a number of apolipoproteins (*APO*s) and negatively correlated with the muscle isozyme of the protein pyruvate kinase (*PKM*). Authors establish a linear model based on a handful of protein quantities that accounts for most variance in α-tocopherol plasma concentration and suggest an inexpensive, portable, antibody-based methodology can be used to assay plasma α-tocopherol abundance in low-income countries [185]. An orbitrap-based study of hippocampi, medial prefrontal cortices, and striata tissue in a mouse model of Alzheimer's disease reports administration of a tocotrienol-rich fraction of palm oil down-regulates hippocampi expression of the amyloid beta A4 protein (*APP*). Amyloid beta A4 is the principle component of amyloid plaques characteristic of Alzheimer's disease [186]. In a QTOF-based study of rabbit aortae, vitamin E supplementation is reported to impact protein abundances including the apolipoprotein, *APOA1*, and several related to oxidation/reduction processes [206]. 2DGE-MS-based studies also report vitamin E supplementation to impact apolipoprotein abundances in human plasma [207, 208]. In a 2DGE-based study of high-density reared rainbow trout livers, vitamin E supplementation is reported to regulate the abundances of a handful of heat shock and metabolic proteins [209]. Finally, a 2DGE-MS-based study reports vitamin E supplementation to regulate a number of plasma protein abundance in patients harboring prostate tumors [210].

### Vitamin K

Vitamin K compounds all share a common fused benzyl and methyl-naphthoquinone ring moiety (Fig. 1). Naturally occurring vitamin K compounds include phylloquinone and menaquinones [211]. Vitamin K is a necessary cofactor of gamma-glutamyl carboxylase (*GGCX*), an enzyme which catalyzes the carboxylation of glutamate protein residues to carboxyglutamate residues [212]. This post-translational modification is necessary for the function of proteins of the coagulation cascade (*F2*, *F7*, *F9*, *F10*), proteins inhibiting coagulation (*PROC*, *PROS1*, *PROZ*), and those associated with connective tissue matrix formation (*BGLAP*, *MGP*) [213]. Newborn infants are among the most at-risk for vitamin K deficiency because they do not have adequate stores and milk is not a sufficient source. Thus a phylloquinone injection shortly after birth is recommended [214]. Elevated risk of hemorrhage is associate with vitamin K deficiency [215].

In an orbitrap-based study of plasma from Nepalese children, authors create a model based on five protein abundances which can predict vitamin K deficiency with moderate accuracy. Vitamin K status is based on a surrogate measurement of an abundance of an abnormal form of prothrombin [187].

## Conclusions

Proteomic studies have established dietary vitamin status as a regulator of tissue protein abundances. The regulatory feedback between vitamin status and protein expression is highlighted by findings where the abundances of proteins directly related to the vitamin are impacted by systemic intake of that vitamin, including: abundances of proteins related to craniofacial development and neural crest processes are impacted in an established maternal retinoic acid toxicity-driven model of craniofacial birth defects [30], deficiency in their riboflavin cofactor is accompanied by reduced abundances of acyl-coenzyme A dehydrogenases and accumulation of the enzymes’ substrates [51], treatment with the active form of vitamin D is accompanied by increased expression of calcium binding proteins [184], and vitamin E supplementation impacts proteins related to redox processes [206] (Table 1). However, the literature in this field is sparse and, in all likelihood, the vast majority of vitamin-status to protein abundance relationships are undescribed; especially considering the void in the literature for several vitamins. Moreover, the field has not advanced to explore the mechanisms of these regulations, their biological impacts, or their potential to shape clinical interventions.

Modern proteomic platforms are ever increasing achievable depth, where analysis of whole mammalian tissue [216–220] or plasma [221, 222] routinely results in 10,000 or 1,500 unique proteins quantified per sample respectively. Furthermore, advances continue in capacity to detect post-translational modifications [223, 224], determine compartmental localization [225], and apply findings in clinical settings [226]. In a golden age of proteomic technological advances, studies of vitamin intake have not kept pace. Of those using orbitrap, QTOF, or triple-TOF systems, many fall short of the cutting edge of analytical depth (Table 2); whereas most studies have relied on antiquated 2DGE-MS platforms. Outdated platforms rendering fewer quantified proteins are likely contributors to clustering [227] or enrichment analysis techniques [228–234] not being widely employed. These high-throughput methods of data analysis provide systems-level stratification of proteome-wide adaptations and can guide targeted inquiries. As the study of precision nutrition advances in an era of big data, fundamental questions of nutrient-protein interactions will be at the forefront of understanding molecular mechanisms of nutrient and substrate processing. Where the sparsity of the literature leaves fundamental questions unanswered, opportunity for rapid advancement lies with application of cutting-edge technologies in well-designed and executed studies.

**Table 2:** Summary of Technical Depth of Orbitrap-, QTOF-, and Triple-TOF-based Studies. A protein only needs to be identified in one sample to contribute to the total number of unique proteins (indicated as “total” below), thus the total is typically larger in studies with greater sample numbers. An iTRAQ or TMT set is a pool of samples that are run on the LC-MS/MS concurrently. Because the samples in a set are all analyzed simultaneously, a set's contribution to the total number of unique proteins is similar to that of a single sample

Study	Year	Platform	Tissue	# Proteins Identified	# Samples
[30]	2018	Orbitrap	mouse embryo heads	group 1: 313 total group 2: 372 total	2 groups
[31]	2018	Triple-TOF	gerbil plasma	109 total	30
[32, 185, 187]	2015	Orbitrap	human plasma	4,705 total 589/set	72 iTRAQ sets
[42]	2018	QTOF	rat thalami	1,440 total	6 x 3 tech. reps.
[51]	2017	QTOF	duck livers	1,749 total	3 iTRAQ sets
[52]	2019	QTOF	fetal duck livers	3,801 total	1 iTRAQ set
[91]	2019	Triple-TOF	rat hippocampi	4,807 total	2 iTRAQ sets
[120]	2015	Orbitrap	human follicular fluid	227 total	1 TMT set
[121]	2014	QTOF	mouse intestine	2,039 total	10
[156]	2019	Orbitrap	pig plasma	534 total	45
[157]	2018	Orbitrap	mouse liver fraction	4,058 total	9
[158]	2014	QTOF	zebrafish	2,956 total	19
[183]	2016	Orbitrap	mouse lung	1,160 total, 240 common to all	34
[184]	2018	Orbitrap	mouse brain	5,062 total	1 TMT set
[188]	2016	Orbitrap	human serum	1,841 total	1 iTRAQ set
[186]	2019	Orbitrap	mouse brain tissues	group 1: 5,847 total group 2: 6,047 total	2 groups of 6
[206]	2013	QTOF	rabbit aortae	100 total	24 x 3 tech. reps.

## Supplementary information

**Additional file 1: Tables S1 - S13.** Ensyme, enzyme complexes, or enzyme families requiring vitamins as a cofactor or substrate.

## Data Availability

All data presented herein is available from the referenced sources.

## Abbreviations

Roman numerals I-IV: Electron transport chain complexes I-IV
2DGE: Two-dimensional gel electrophoresis
2DGE-MS: 2DGE – mass spectrometry
3PG: 3-phosphoglycerate
6PG: 6-phosphogluconate
6PGL: 6-phosphogluconolactone
AcAc: Acetoacetate
AcCoA: Acetyl-CoA
ADP: Adenosine diphosphate
aKG: Alpha ketoglutarate
Ala: Alanine
Asp: Aspartate
ATP: Adenosine triphosphate
CoA: Coenzyme A
Cit: Citrate
CTP: Cytidine triphosphate
DHO: Dihydroorotate
DNA: Deoxyribonucleic acid
dTMP: Deoxythymidine monophosphate
dUDP: Deoxyuridine diphosphate
dUMP: Deoxyuridine monophosphate
F6P: Fructose 6-phosphate
Fum: Fumarate
G3P: Glyceraldehyde 3-phosphate
G6P: Glucose 6-phosphate
Glc: Glucose
GlcN: Glucosamine
GlcNAc: N-acetylglucosamine
Gln: Glutamine
Glu: Glutamate
Gly: Glycine
Hcy: Homocysteine
HIF1a: Hypoxia inducible factor 1 subunit alpha
HMGCoA: 3-hydroxy-3-methylglutaryl-CoA
IC: Isocitrate
Ile: Isoleucine
Leu: Leucine
Mal: Malate
mDNA: Methylated DNA
Met: Methionine
MMCoA: Methylmalonyl-CoA
Oac: Oxaloacetate
PEP: Phosphoenolpyruvate
PPCoA: Propionyl-CoA
Pyr: Pyruvate
QTOF: Quadrupole time-of-flight
QQQ: Triple-quadrupole
R5P: Ribose 5-phosphate
reps.: Replicates
Ru5P: Ribulose 5-phosphate
SAH: S-adenosylhomocysteine
SAM: S-adenosylmethionine
Ser: Serine
Suc: Succinate
SucCoA: Succinyl-CoA
tech.: Technical
THF: Tetrahydrofolate
Thr: Threonine
UDP: Uridine diphosphate
UTP: Uridine triphosphate
UQ: Ubiquinone
Val: Valine
X5P: Xylulose 5-phosphate
