# Supplementary material for: Systemic vitamin intake impacting tissue proteomes
Source: Nutr Metab (Lond). 2020 Aug 26;17:73. doi: 10.1186/s12986-020-00491-7 (PMC7449053; doi:10.1186/s12986-020-00491-7)
Supplement: Supplementary file 1 — Additional file 1: Tables S1 - S13. Ensyme, enzyme complexes, or enzyme families requiring vitamins as a cofactor or substrate. [file 12986_2020_491_MOESM1_ESM.docx]

**Table S1: Enzyme, enzyme complexes, or enzyme families requiring vitamin A as a cofactor or substrate**

| **Enzyme** | **Function** |
| --- | --- |
| Rhodopsin kinase (*GRK1*) | Phosphorylation of rhodopsin to terminate phototransduction. |
| Retinoic acid binding proteins (*CRABP1*, *CRABP2*) | Solubilization of retinoic acid and regulation of intracellular retinoic acid metabolism. |
| Retinol binding protein (*RBP1*, *RBP2*, *RBP3*, *RBP4*, *RBP5*) | Transport of retinol from hepatic parenchymal cell into the blood by forming holo-retinol binding protein (holo-RBP). |
| Transthyretin (*TTR*) | Transport of holo-RBP in the blood by forming holo-RBP-transthyretin ternary complex. |
| β-carotene 9’10’ dioxygenase (*BCO2*) | Cleavage of β-carotene into β-apo-10’-carotenal and β-ionine. |
| β-carotene 15,15’-dioxygenase (*BCO1*) | Cleavage of β-carotene into retinal. |
| Lecithin retinol acyltransferase (*LRAT*) | Esterification of retinol by transferring *sn*-1 fatty acids from phosphatidylcholine to retinol. |
| Retinol dehydrogenases (*RDH5*, *RDH8*, *RDH10*, *RDH11*, *RDH12*, *RDH13*, *RDH14*, *RDH16*, *DHRS1*, *DHRS2*, *DHRS3*, *DHRS4*, *DHRS4L1*, *DHRS4L2*, *DHRS7*, *DHRS7B*, *DHRS7C*, *DHRS9*, *DHRS11*, *DHRS12*, *DHRS13*, *DHRSX*) | Conversion of retinol to retinal. |

**Table S2: Enzyme, enzyme complexes, or enzyme families requiring vitamin B1 (thiamine) as a cofactor or substrate**

| **Enzyme** | **Function** |
| --- | --- |
| Pyruvate dehydrogenase complex (*PDHA1*, *PDHA2*, *PDHB*, *PDHX*, *DLAT*, *DLD*) | Decarboxylation of pyruvate to acetyl-CoA. |
| α-Ketoglutarate dehydrogenase complex (*OGDH*, *DLST*, *DLD*) | Decarboxylation of α-ketoglutarate to succinyl-CoA. |
| Branched-chain α-keto acid dehydrogenase complex (*BCKDHA*, *BCKDHB*, *DBT*, *DLD*) | Transamination of valine, isoleucine and leucine. |
| Transketolase (*TKT*) | Synthesis of pentoses and NADPH in glycolysis and pentose phosphate pathway. |
| Thiamine transporters (*SLC19A2*, *SLC19A3*) | Transport of thiamine across the plasma membrane. |
| Mitochondrial thiamine pyrophosphate carrier (*SLC25A19*) | Transport of thiamine pyrophosphate into mitochondria. |

**Table S3: Enzyme, enzyme complexes, or enzyme families requiring vitamin B2 (riboflavin) as a cofactor or substrate**

| **Enzyme** | **Function** |
| --- | --- |
| Succinate dehydrogenase complex (*SDHA*, *SDHB*, *SDHC*, SDHD) | Complex II in electron transport chain.  Conversion of succinate to fumarate in TCA cycle. |
| Pyruvate dehydrogenase complex (*PDHA1*, *PDHA2*, *PDHB*, *PDHX*, *DLAT*, *DLD*) | Decarboxylation of pyruvate to acetyl-CoA. |
| *sn*-Glycerophosphate dehydrogenase (*GPD2*) | NADH shuttle between glycolysis and electron transport chain in mitochondria. |
| Electron transfer flavoprotein dehydrogenase (*ETFDH*) | Transfer electron from electron transfer flavoprotein to ubiquinone. |
| Dihydrolipoyl dehydrogenase (*DLD*) | Oxidative decarboxylation of pyruvate by pyruvate dehydrogenase complex. |
| Acyl-CoA dehydrogenases (*ACADM*, *IVD*, *ACADS*, *ACADL*, *GCDH*, *ACADVL*, *ACADSB*, *ACAD8*, *ACAD9*, *ACAD10*, *ACAD11*) | Dehydrogenation of acyl-CoA esters in fatty acid β-oxidation. |
| Dimethylglycine dehydrogenase (*DMGDH*) | Catalysis of dimethylglycine to form sarcosine in choline catabolism. |
| Choline dehydrogenase (*CHDH*) | Choline catabolism. |
| Sarcosine dehydrogenase (*SARDH*) | Catalysis of sarcosine to form glycine. |
| NADPH oxidases (*NOX1*, *NOX2*, *NOX3*, *NOX4*, *NOX5*, *DUOX1*, *DUOX2*) | Production of superoxide and hydroperoxide. |
| Xanthine oxidase (*XDH*) | Oxidation of hypoxanthine to xanthine in purine catabolism. |
| Aldehyde oxidase (*AOX1*) | Aldehyde oxidation in pyridoxic acid and retinoic acid metabolism. |
| Pyridoxine phosphate oxidase (*PNPO*) | Conversion of pyridoxamine phosphate to pyridoxal phosphate, an active form of vitamin B_6_. |
| D- and L-amino oxidase (*DAO*, *IL4IA*) | Oxidative deamination of D- and L-amino acids. |
| Monoamine oxidase (*MAOA*, *MAOB*) | Dopamine, tyramine and histamine metabolism. |
| Sulfhydryl oxidases (*QSOX1*) | Disulfide bond formation in proteins. |
| Spermine oxidase (*SMOX*) | Amine catabolism. |
| Kynurenine 3-monooxygenase (*KMO*) | Conversion of kynurenine to 3-hydroxy-kynurenine in tryptophan metabolism. |
| Squalene monooxygenase (*SQLE*) | Squalene oxidation in cholesterol synthesis. |
| Flavin monooxygenases (*FMO1*, *FMO2*, *FMO3*, *FMO4*, *FMO5*, *FMO6*) | Oxidation of amines and sulfides. |
| NADPH-cytochrome P450 oxidoreductase (*POR*) | Electron donor for cytochrome *b_5_*, heme oxygenase, 3-β-hydroxysterol delta-24-reductase and squalene monooxygenase. |
| NADH-ubiquinone oxidoreductase (*NDUFV1*, *NDUFV2*, *NDUFV3*) | Complex I in electron transport chain. |
| Cytochrome *b_5_* reductase (*CYB5R1*, *CYB5R2*, *CYB5R3*, *CYB5R4*) | Double bond formation in fatty acids. |
| 3-β-Hydroxysterol delta-24-reductase (*DHCR24*) | Conversion of desmosterol to cholesterol. |
| 7-Dehydrocholesterol reductase (*DHCR7*) | Conversion of 7-dehydrocholesterol to cholesterol. |
| Methylenetetrahydrofolate reductase (*MTHFR*) | Conversion of 5,10-methylene tetrahydrofolate to 5-methyl tetrahydrofolate. |
| Glutathione reductase (*GSR*) | Reduction of glutathione disulfide (GSSG) to glutathione (GSH). |
| Thioredoxin reductase (*TXNRD1*, *TXNRD2*, *TXNRD3*) | Thioredoxin reduction in conversion of ribonucleotides to deoxyribonucleotides in DNA synthesis. |
| 3-Ketosphinganine reductase (*KDSR*) | Conversion of 3-ketosphinganine to sphinganine in sphingosine synthesis. |
| Dihydroceramide desaturase (*DEGS1*, *DEGS2*) | Desaturation of dihydroceramide to maintain balance between sphingolipids and dihydrosphingolipids. |
| Dihydroxyacetone phosphate acyltransferase (*GNPAT*) | Ether lipids synthesis in peroxisomes. |
| Alkyl dihydroxyacetone phosphate synthase (*AGPS*) |  |
| FAD synthase (*FLAD1*) | Adenylation of flavin mononucleotide (FMN) for flavin adenine dinucleotide (FAD) generation. |
| Nitric oxide synthases (*NOS1*, *NOS2*, *NOS3*) | Production of nitric oxides from L-arginine. |
| Lysine-specific demethylase 1 (*KDM1A*) | Demethylation of mono- and di-methylated lysine 4 of histone H3 (H3K4) and lysine 9 of histone 3 (H3K9). |

**Table S4: Enzyme, enzyme complexes, or enzyme families requiring vitamin B3 (niacin) as a cofactor or substrate**

| **Pathway** | **Enzyme** |
| --- | --- |
| Glycolysis | Glyceraldehyde-3-phosphate dehydrogenase (*GAPDH*) |
| Interconversion of pyruvate and lactate | Lactate dehydrogenase (*LDHA*, *LDHB*, *LDHC*) |
| TCA cycle | Pyruvate dehydrogenase (*PDHA1*, *PDHA2*, *PDHB*), Isocitrate dehydrogenase (*IDH1*, *IDH2*, *IDH3A*, *IDH3B*, *IDH3G*), α-Ketoglutarate dehydrogenase (*OGDH*), Malate dehydrogenase (*MDH1*, *MDH2*) |
| Fatty acid β-oxidation | β-Hydroxyacyl-CoA dehydrogenase (*HADH*, *HSD17B10*, *HSD17B4*, *EHHADH*) |
| Vitamin B_6_ Catabolism | Aldehyde dehydrogenases (*ALDH3A1*, *ALDH3A2*, *ALDH3B1*, *ALDH3B2*, *ALDH1A1*, *ALDH1A2*, *ALDH1A3*, *ALDH1B1*, *ALDH1L1*, *ALDH1L2*, *ALDH2*, *ALDH4A1*, *ALDH5A1*, *ALDH6A1*, *ALDH7A1*, *ALDH8A1*, *ALDH9A1*, *ALDH16A1*, *ALDH18A1*) |
| Pentose phosphate pathway | Glucose-6-phosphate dehydrogenase (*G6PD*), 6-Phosphogluconate dehydrogenase (*PGD*) |
| Malate aspartate shuttle | Malate dehydrogenase (*MDH1*, *MDH2*) |
| Glycerol-3-phosphate shuttle | Glycerol-3-phosphate dehydrogenase (*GPD1*, *GPD2)* |
| Fatty acid and steroid synthesis | β-Ketoacyl-acyl carrier protein (ACP) reductase (*HSD17B8*), 3-Hydroxy-3-methylglutaryl coenzyme A (HMG-CoA) reductase (*HMGCR*) |
| Triacylglycerol synthesis | Diacylglycerol O-transferase 1 (*DGAT1*) |
| Ketone body formation | 3-Hydroxybutyrate dehydrogenase (*BDH1*, *BDH2*) |
| Folate-dependent one carbon metabolism | Dihydrofolate reductase (*DHFR*, *DHFR2*), Methylene THF reductase (*MTHFR*), Methylene THF dehydrogenase (*MTHFD1*, *MTHFD2*) |
| Branched chain amino acid catabolism | β-Hydroxyisobutyrate dehydrogenase (*HIBADH*), Methylmalonic semialdehyde dehydrogenase (*ALDH6A1*), Branched chain keto acid dehydrogenase (*BCKDHA*, *BCKDHB*), β-Hydroxyacyl-CoA dehydrogenase (*HADH*, *HSD17B10*, *HSD17B4*, *EHHADH*) |
| Lysine catabolism | Lysine-α-ketoglutarate reductase (*AASS*), Saccharopine dehydrogenase (*SCCPDH*), L-α-Aminoadipate-δ-semialdehyde dehydrogenase (*AASDH*) |
| Serine and glycine metabolism | D-Glycerate dehydrogenase (*GRHPR*), D-3-Phosphoglycerate dehydrogenase (*PHGDH*) |
| Pyrimidine synthesis | Dihydroorotate dehydrogenase (*DHODH*), Ribonucleotide reductase (*RRM1*, *RRM2*, *RRM2B*) |
| ADP-ribosylation reactions for post translational modifications | ADP ribose polymerases (*PARP1*, *PARP2*, *PARP3*, *PARP4*, *TNKS*, *TNKS2*, *PARP6*, *TIRARP*, *PARP8*, *PARP9*, *PARP10*, *PARP11*, *PARP12*, *PARP13*, *PARP14*, *PARP15*, *PARP16*), ADP-ribosyl transferases (*ART1*, *ART3*, *ART4*, *ART5*) |
| Synthesis of cyclic adenosine monophosphate (cAMP) for intracellular signal transduction | Adenylate cyclases (*ADCY1*, *ADCY2*, *ADCY3*, *ADCY4*, *ADCY5*, *ADCY6*, *ADCY7*, *ADCY8*, *ADCY9*, *ADCY10*) |
| Cyclic ADP-ribose and nicotinic acid adenine dinucleotide phosphate (NAADP) formation in calcium metabolism | ADP-ribosyl cyclases (*CD38*, *BST1*) |
| Sirtuin deacetylation in p53 function and genomic stability | NAD-dependent deacetylases sirtuins 1-6 (*SIRT1*, *SIRT2*, *SIRT3*, *SIRT4*, *SIRT5*, *SIRT6*) |

**Table S5: Enzyme, enzyme complexes, or enzyme families requiring vitamin B5 (pantothenic acid) as a cofactor or substrate**

| **Enzyme** | **Function** |
| --- | --- |
| 10-Formylteterahydrofolate dehydrogenase (*ALDH1L1*, *ALDH1L2*) | Oxidation of 10-formylteterahydrofolate to form tetrahydrofolate. |
| Cytosolic fatty acid synthase (*FASN*) | Fatty acid synthesis. |
| 4’-Phosphopantetheine transferase (*AASDHPPT*) | Addition of 4’-phosphopantetheine to proteins. |
| α-Aminoadipate semialdehyde dehydrogenase (*ALDH7A1*) | Conversion of lysine to α-aminoadipate semialdehyde in lysine degradation. |
| Pyruvate dehydrogenase complex (*PDHA1*, *PDHA2*, *PDHB*, *PDHX*, *DLAT*, *DLD*) | Decarboxylation of pyruvate to acetyl-CoA. |
| α-Ketoglutarate dehydrogenase complex (*OGDH*, *DLST*, *DLD*) | Decarboxylation of α-ketoglutarate to succinyl-CoA. |
| Branched-chain α-keto acid dehydrogenase complex (*BCKDHA*, *BCKDHB*, *DBT*, *DLD*) | Transamination of valine, isoleucine and leucine. |

**Table S6: Enzyme, enzyme complexes, or enzyme families requiring vitamin B6 as a cofactor or substrate**

| **Enzyme** | **Function** |
| --- | --- |
| Aminotransferases | Transfer of an amino group from an amino acid to α-keto acid in nonessential amino acids synthesis. Aspartic amino transferase (*GOT1*, *GOT2*) and alanine aminotransferase (*GPT*, *GPT2*) are the common aminotransferases with vitamin B_6_ as coenzymes. |
| Decarboxylases | Removal of carboxy group from amino acids in the formation of γ-aminobutyric acid (glutamate decarboxylase (*GAD1*, *GAD2*)), serotonin (*DDC*), histamine, dopamine and epinephrine. |
| Racemases (*SRR*) | Interconversion of D-amino acids and L-amino acid. |
| Dehydratases (*SDS*) | Removal of an amino group from amino acids. |
| Cystathionine β-synthase (*CBS*) | Conversion of homocysteine to cystathionine in cysteine synthesis. |
| Cystathionine γ-lyase (*CTH*) | Conversion of cystathionine to cysteine. |
| Selenocysteine β-lyase (*SCLY*) | Conversion of selenocysteine to selenide. |
| Serine hydroxymethyltransferase (*SHMT1*, *SHMT2*) | Transfer of hydroxyl group from serine to tetrahydrofolate (THF) for glycine generation. |
| δ-Aminolevulinate synthase (*ALAS1*, *ALAS2*) | Labilization of glycine and addition of succinate to generate δ-aminolevulinate, the first and rate-limiting step in heme synthesis. |
| Glycogen phosphorylase (*PYGM*, *PYGL*) | Degradation of glycogen to glucose-1-phosphate. |
| Serine palmitoyl transferase (*SPTLC1*, *SPTLC2*, *SPTLC3*) | Condensation of serine with palmitoyl-CoA in sphingolipid synthesis. |
| δ-6-Desaturase (*FADS2*) | Desaturation of linoleic and γ-linolenic acids. |
| Kynureninase (*KYNU*) | Conversion of 3-hydroxykynurenine to 3-hydroxyanthranilic acid in niacin synthesis. |

**Table S7: Enzyme, enzyme complexes, or enzyme families requiring vitamin B7 (biotin) as a cofactor or substrate**

| **Enzyme** | **Function** |
| --- | --- |
| Pyruvate carboxylase (*PC*) | Carboxylation of pyruvate to oxaloacetate in anaplerotic reaction. |
| Acetyl-CoA carboxylase (*ACACA*, *ACACB*) | Carboxylation of acetyl-CoA to malonyl-CoA. |
| Propionyl-CoA carboxylase (*PCCA*, *PCCB*) | Carboxylation of propionyl-CoA generated from catabolism of odd-chain fatty acids, isoleucine, threonine and methionine to form methylmalonyl-CoA. |
| β-Methylcrotonyl-CoA carboxylase (*MCCC1*, *MCCC2*) | Carboxylation of β-methylcrotonyl-CoA generated from catabolism of leucine to form β-methylglutaconyl-CoA which can be further catabolized to acetoacetate and acetyl-CoA. |
| Holocarboxylase synthetase (*HLCS*) | Catalysis of covalent linkage of biotin to lysine residues of carboxylases. |
| Sodium-dependent solute carriers (*SLC5A6*, *SLC19A3*) | Transport of biotin across the plasma membrane |

**Table S8: Enzyme, enzyme complexes, or enzyme families requiring vitamin B9 (folic acid) as a cofactor or substrate**

| **Enzyme** | **Function** |
| --- | --- |
| Glycinamide ribonucleotide formyltransferase (*GART*) | Addition of formyl groups at C8 position in 10-formyl THF in purine synthesis. |
| 5-amino-4-imidazole carboxamide ribonucleotide formyltransferase (*ATIC*) | Addition of formyl groups at C2 position in 10-formyl THF in purine synthesis. |
| Thymidylate synthetase (*TYMS*) | Conversion of deoxyuridine monophosphate (dUMP) to deoxythymidine monophosphate (dTMP), a rate-limiting step in DNA synthesis. |
| Serine hydroxymethyltransferase (*SHMT1*, *SHMT2*) | Conversion of THF and serine to 5,10-methylene THF and glycine. |
| Methionine synthase (*MTR*) | Conversion of 5-methyl THF and homocysteine to THF and methionine. |
| 10-Formyl THF synthetase (*MTHFD1*, *MTHFD1L*) | Formylation of THF to form 10-Formyl-THF. |
| 5,10-Methenyl THF cyclohydrolase (*MTHFD1*, *MTHFD2*) | Conversion of 10-formyl THF to 5,10-methenyl THF. |
| 5,10-Methylene-THF dehydrogenase (*MTHFD1*, *MTHFD2*) | Conversion of 5,10-methenyl THF to 5,10-methylene THF. |
| Dihydrofolate reductase (*DHFR*, *DHFR2*) | Conversion of folic acid to dihydrofolate.  Conversion of dihydrofolate to THF. |
| Methylene THF reductase (*MTHFR*) | Conversion of 5,10-methylene THF to 5-methyl THF |

**Table S9: Enzyme, enzyme complexes, or enzyme families requiring vitamin B12 (cobalamin) as a cofactor or substrate**

| **Enzyme** | **Function** |
| --- | --- |
| Methionine synthase (*MTR*) | Conversion of homocysteine to methionine in the cytosol with methylcobalamin as a cofactor. |
| Methylmalonyl-CoA mutase (*MUT*) | Isomerization of methylmalonyl-CoA to succinyl-CoA to catabolize odd-chained fatty acids and cholesterol in the mitochondria with adenosylcobalamin as a cofactor. |

**Table S10: Enzyme, enzyme complexes, or enzyme families requiring vitamin C (ascorbic acid) as a cofactor or substrate**

| **Enzyme** | **Function** |
| --- | --- |
| Dopamine β-hydroxylase (*DBH*) | Hydroxylation of dopamine to form norepinephrine. |
| Peptidylglycine α-amidating monooxygenase (*PAM*) | Addition of carboxy-terminal α-amide group to activate peptide hormones. |
| Prolyl 3-hydroxylase (*P3H1*, *P3H2*, *P3H3*) | Hydroxylation of prolyl and lysyl residues in collagen synthesis. |
| Prolyl 4-hydroxylase (*P4HA1*, *P4HA2*, *P4HA3*, *P4HB*, *P4HTM*) |  |
| Lysyl hydroxylase (*PLOD1*, *PLOD2*, *PLOD3*) |  |
| Hypoxia inducible factor prolyl hydroxylases (*EGLN1*, *EGLN2*, *EGLN3*) | Hydroxylation of hypoxia inducible factor 1 subunit alpha (HIF1A) protein. |
| Tyrosine hydroxylase (*TH*) | Conversion of tyrosine to L-DOPA in catecholamine biosynthesis. |
| Trimethyllysine hydroxylase (*TMLH*) | Conversion of trimethyllysine to hydroxy trimethyllysine in carnitine biosynthesis. |
| γ-Butyrobetaine hydroxylase (*BBOX*) | Conversion of γ-butyrobetaine to carnitine in carnitine biosynthesis. |
| 4-Hydroxyphenylpyruvate dioxygenase (*HPD*) | Conversion of 4-hydroxyphenylpyruvate to homogentisate in tyrosine catabolism. |
| Sodium-dependent vitamin C transporters (*SLC23A1*, *SLC23A2*) | Absorption of ascorbic acid at the intestinal brush border and distribution to tissues. |
| Glucose transporters (*SLC2A1*, *SLC2A3*, *SLC2A4*) | Absorption of oxidized ascorbic acid at the gastrointestinal tract and distribution to tissues. |
| Carnitine-acylcarnitine translocase (*SLC25A20*) | Transport of carnitine fatty acid complexes into inner mitochondria. |

**Table S11: Enzyme, enzyme complexes, or enzyme families requiring vitamin D as a cofactor or substrate**

| **Enzyme** | **Function** |
| --- | --- |
| Vitamin D binding protein (*GC*) | Transport of vitamin D metabolites in the blood to tissues. |
| Protein disulfide isomerase A3 (*PDIA3*) | Regulation of rapid membrane-associated signaling pathway of vitamin D. |
| 1α-hydroxylase (*CYP27B1*) | Conversion of 25(OH)D to 1,25(OH)_2_D_3_ in the kidney. |
| 24-hydroxylase (*CYP24A1*) | Degradation of 1,25(OH)_2_D_3_ and 25(OH)D to inactive forms. |
| 25-hydroxylase (*CYP2R1*) | Conversion of vitamin D_3_ to 25(OH)D_3_. |

**Table S12: Enzyme, enzyme complexes, or enzyme families requiring vitamin E as a cofactor or substrate**

| **Enzyme** | **Function** |
| --- | --- |
| α-tocopherol transfer protein (*TTPA*) | Transfer of RRR α-tocopherol and to very low-density lipoprotein (VLDL) for distribution to tissues. |

**Table S13: Enzyme, enzyme complexes, or enzyme families requiring vitamin K as a cofactor or substrate**

| **Enzyme** | **Function** |
| --- | --- |
| γ-Glutamyl carboxylase (*GGCX*) | Posttranslational carboxylation of glutamic acid residues to form γ-carboxy-glutamate required for coagulation and bone mineralization. |
